# Supplementary material for: A New Defective Helper RNA to Produce Recombinant Sindbis Virus that Infects Neurons but does not Propagate
Source: Front Neuroanat. 2016 May 24;10:56. doi: 10.3389/fnana.2016.00056 (PMC4877524; doi:10.3389/fnana.2016.00056)
Supplement: Supplementary file 1 [file DataSheet_1.docx]

dir1=getDirectory("Please select the directory where images are stored in");

dir2=getDirectory("Please select the directory where Result are stored in");

list=getFileList(dir1); // Get all files in dir1

for (i=0;i<list.length;i++)

{

if (endsWith(list[i], ".czi")) // Only process .czi file. All other files will be ingored.

{

temp=dir1+list[i];

run("Bio-Formats Windowless Importer", "open=[temp]");

run("Set Scale...", "distance=0 global");

makeRectangle(0, 0, 1320, 1040); // Crop

run("Crop");

name0 = getTitle;

index = lastIndexOf(name0, ".");

if (index!=-1) name = substring(name0, 0, index);

saveAs("Jpeg", dir2+name+"1st"); // Original picture, save for reference

run("Despeckle"); //delete noise

run("Gaussian Blur...", "sigma=2");

run("8-bit"); // Convert it into 8-bits picture.

setAutoThreshold("Default dark");

setThreshold(12, 255); //Threshold

run("Threshold...");

run("Convert to Mask");

run("Fill Holes");

saveAs("Jpeg", dir2+name+"2nd"); // Threshould picture, save for reference.

run("Analyze Particles...", "size=8-Infinity show=Outlines display clear"); // Min area is 8. Smaller than 8 is considered as noise.

saveAs("Jpeg", dir2+name+"3rd"); // Acutal cells being counted, save for reference.

close();

selectWindow("Results");

name = name + ".csv";

saveAs("Measurements", dir2+name);

close();

}

}
